# Supplementary material for: Measuring the Whole Wall Thickness of the Common Femoral Vein as a Distinctive Diagnostic Tool to Distinguish Behçet’s Disease Presenting with Oral Ulcers from Recurrent Aphthous Stomatitis
Source: Diagnostics (Basel). 2023 Aug 19;13(16):2705. doi: 10.3390/diagnostics13162705 (PMC10453469; doi:10.3390/diagnostics13162705)
Supplement: Supplementary file 1 [file diagnostics-13-02705-s001.zip › diagnostics-2525908-supplementary.pdf]

Supplementary Table S1. Summary of Doppler ultrasound studies on vein wall and intima-media thickness measurements in BD

Author (reference)	Study population	Which veins were studied	Findings	Conclusion
Alibaz-Oner et al. (12)	Healthy male control (n = 37)<br>Male patients with BD (n = 61)<br>Male patients with AS (n = 27)	Common femoral vein<br>Great/Small saphenous vein	All venous measurements were significantly higher in BD compared to AS and HC. Both right and left extremity CFV thicknesses had a high area under the ROC curve (> 0.8). Cut-off values for right and left CFV thicknesses for BD was 0.49 and 0.48 mm, respectively. High sensitivity and specificities are observed for both measurements.	Increased CFV thickness was found in BD patients independent of vascular involvement. As a similar change was not observed in controls, increased CFV thickness may be a specific sign of venous inflammation in BD.
Seyahi et al. (13)	Healthy control (n = 50)<br>BD patients with LE DVT (n = 50)<br>BD patients without any vascular involvement (n = 50)	Common femoral vein<br>Femoral vein<br>Great saphenous vein	The mean VWT was significantly increased in both BD patients with LE DVT and those without apparent vascular involvement compared with the healthy controls, whereas those with LE DVT had the highest VWT.	VWT of proximal deep and superficial LE veins is increased among the BD patients without any clinical and radiologic vascular involvement.
Alibaz-Oner et al. (14)	Healthy control (n = 38)<br>Patients with BD (n = 69)<br>Patients with CD (n = 38)	Common femoral vein	Both right and left CFV thicknesses were significantly higher in BD compared to HC and CD. CFV thicknesses in CD were similar to HC. CFV thickness was also similar between BD patients with and without GI involvement. The diagnostic cutoff values of ≥ 0.5 mm for CFV thickness performed well against to both CD and HC for discrimination of BD. The sensitivity and specificity rates were > 85% for both HC and CD. Positive and negative predictive values were > 90%.	A significantly lower CFV thickness was found in CD compared to BD. CFV wall thickness measurement is a distinctive diagnostic tool for the differentiation of BD and CD and can be helpful in daily practice for the differentiation of two diseases.
Alibaz-Oner et al. (15)	Healthy controls (n = 51)<br>Patients with BD (n = 152)<br>Patients with AS (n = 27)<br>Patients with systemic vasculitides (n = 23)<br>Patients with venous insufficiency (n = 29)<br>Patients with APS (n = 43)<br>Patients with DVT due to non-inflammatory causes (n = 25)	Common femoral vein	Bilateral CFV thickness was significantly increased in BD compared with all control groups. The area under the receiver operating characteristic curve for bilateral CFV thicknesses in all comparator groups was >0.95 for the cut-off value (0.5 mm). This cut-off value also performed well against all control groups with sensitivity rates >90%. The specificity rate was also >80% in all comparator groups except APS (positive predictive value: 79.2–76.5%, negative predictive value: 92–91.8% for right and left CFV, respectively).	Increased CFV thickness is a distinctive feature of BD and is rarely present in healthy and diseased controls, except APS. CFV thickness measurement with ultrasonography can be a diagnostic tool for BD with sensitivity and the specificity rates higher than 80% for the cut-off value ≥0.5 mm.

Kaymaz et al. (16)	Healthy control (n = 30)<br>Patients with BD (n = 63)	Carotid intima media<br>Jugular vein<br>Common femoral vein<br>Main portal vein	All VWTs and CIMT were significantly higher in patients with BD compared to healthy controls. There was no significant difference between the BD groups in terms of CIMT, jugular and common femoral VWT. But portal VWT was significantly higher in patients with vascular involvement. A cut-off value of ≥1.35 mm yielded a sensitivity of 79.2% and a specificity of 82.4% for the diagnosis of vascular involvement with the highest Youden's index.	Portal VWT has high sensitivity and specificity for the screening of vascular involvement in patients with BD.
Sevik et al. (17)	Healthy control (n = 41)<br>Patients with BD (n = 53)	Common femoral vein (Whole wall and intima-media thicknesses)	İncreased IMT of CFV was found compared to controls as well as CFV wall thickness.	This study shows that there is a full layer venous wall inflammation in BD independent of vascular involvement. Venous endothelial inflammation may trigger the thickening of the vein wall and cause thrombotic tendency in BD.
Tezcan et al. (11)	Healthy control (n = 52)<br>Patients with BD (n = 54)	Common femoral vein<br>Great saphenous vein<br>Popliteal vein	Venous wall thickness of the lower extremity veins was higher in the BD group and higher in those with a history of deep vein thrombosis than in those without. The mean leukocyte, monocyte, ESR, CRP, PCT, RDW, MPV values, and MLR were higher in BD patients than in the control group. There was a correlation among increased VWT, ESR, PCT, MPV, RDW, and MLR.	CRP, ESR, MPV, PCT, MLR, RDW, and VWT can be used to assist in the diagnosis of BD.
Atalay et al. (18)	Pediatric healthy control (n = 27)<br>Pediatric patients with BD (13 met the criteria for the diagnosis of BD, 22 incomplete BD) (n = 35)	Common femoral vein<br>Femoral vein<br>Vena saphena magna<br>Vena saphena parva<br>Popliteal vein	The median VWT values of both definite and incomplete BD patients were significantly higher than the control group in all veins on both sides. Regarding the best cut-off values of VWT for all lower extremity veins, the sensitivity rates were between 63% and 86%, while specificity rates were between 71% and 100%.	Increased VWT was present not only in BD patients with vascular involvement but also in those without.<br>VWT may be a new criterion in supporting the diagnosis of childhood BD both in definite and incomplete BD patients.

Abbreviations: BD: Behçet disease, HC: Healthy control, LE: Lower extremity, DVT: Deep venous thrombosis, VWT: Vein wall thickness, AS: Ankylosing spondylitis, CFV: Common femoral vein, FV: Femoral vein, CD: Crohn's disease, APS: Antiphospholipid syndrome, CIMT: Carotid intima-media thickness, IMT: İntima-media thickness, ESR: Erythrocyte sedimentation rate, CRP: C-reactive protein, PCT: Plateletcrit, RDW: Red cells distribution width, MPV: Mean platelet volume, MLR: Monocyte to lymphocyte ratio.
